# Prediction of total iodine dose of I-131 therapy for Graves’ hyperthyroidism achieved remission status: a random forest regressor model approach to assess treatment efficacy

**DOI:** 10.3389/fendo.2025.1729926

**Published:** 2026-01-02

**Authors:** Lu Lu, Dongyun Meng, Xiaojuan Wei, Yan Chen, Shaozhou Mo, Zeyong Sun, Fengyang Song, Yuehua Li, Kehua Liao, Wentan Huang

**Affiliations:** Department of Nuclear Medicine, The People’s Hospital of Guangxi Zhuang Autonomous Region, Nanning, Guangxi Zhuang Autonomous Region, China

**Keywords:** dose prediction, Graves’ hyperthyroidism, iodine-131 therapy, random forest regressor, SHAP

## Abstract

**Objective:**

Graves’ hyperthyroidism (GH) presents significant challenges in optimizing Iodine-131 (I-131) therapy, largely due to the variability in patient responses and the limitations of traditional dosing methods. This study aimed to develop and validate a Random Forest Regressor (RFR) model to predict the effective total iodine dose (TID) necessary to achieve remission in patients with GH, thereby enhancing precision and individualization in patient management.

**Methods:**

A retrospective cohort study design was employed, analyzing comprehensive clinical data from 975 adult GH patients who achieved remission and underwent ^131^I therapy 25 January 2015 and 8 August 2023. The cohort, consisting of 975 patients, was divided into a development set (n = 633, spanning from 25 January 2015 to 25 January 2021) and a temporal validation set (n = 342, covering the period from 26 January 2021 to 8 August 2023). A RFR model was developed, utilizing variables such as gender, iodine dose per gram of thyroid tissue (IDPG), Free Thyroxine (FT4), 24-hour Radioactive Iodine Uptake (RAIU24h), Effective half-life (Teff), and thyroid weight to predict the TID. The model’s interpretability was further enhanced using SHapley Additive exPlanations (SHAP) values.

**Results:**

Key predictive variables identified through LASSO-Gaussian regression analysis were gender, IDPG, FT4, RAIU24h, Teff, and thyroid weight. The RFR model demonstrated strong predictive performance, achieving an R-squared value of 0.858 ± 0.05 on the validation set and 0.838 on the temporal validation set, indicating its high capability to explain the variance in TID. SHAP analysis provided crucial insights into the contribution of each feature, highlighting, for example, that high FT4, Teff, and thyroid weight were primary positive contributors to the predicted TID, while RAIU24h offered a compensatory negative contribution.

**Conclusion:**

In conclusion, this study successfully developed and validated an RFR model that accurately predicts the TID for GH patients achieved remission. By integrating multi-dimensional features and providing interpretability through SHAP values, this model offers a sophisticated approach to dose personalization. This advancement has the potential to significantly improve ^131^I treatment efficacy, minimize adverse effects such as hypothyroidism, and foster more precise, individualized patient care in GH.

## 1 Introduction

Graves’ hyperthyroidism (GH) is a prevalent autoimmune thyroid disorder, primarily characterized by the overproduction of thyroid hormones and a multi-system hypermetabolic syndrome. The global prevalence of GH is estimated to be between 0.5% and 2%, with a markedly higher incidence in females compared to males, reflected in a male-to-female ratio of approximately 1:5-10 (1). This disease is most commonly seen in individuals aged 30-50, but its age distribution is wide, and it can also occur in adolescents and the elderly (2). The clinical manifestations of GH are diverse, with typical symptoms including palpitations, weight loss, excessive sweating, anxiety, tremors, and thyroid enlargement (3). Some patients may also have Graves’ orbitopathy, presenting as exophthalmos, diplopia, and visual impairment, severely affecting quality of life (4). If not treated promptly and effectively, GH can lead to a series of serious complications, such as thyroid storm, heart failure, osteoporosis, and arrhythmias, which can even be life-threatening (5, 6). Furthermore, the long-term state of hyperthyroidism has significant negative impacts on patients’ mental health and social functioning, highlighting the importance of its clinical management.

Iodine-131 (^131^I) therapy is one of the important treatment methods for GH, especially suitable for patients with poor efficacy, intolerance, or recurrence of antithyroid drugs (ATD) (7). The treatment principle is to selectively destroy thyroid follicular cells using the β rays released by the decay of ^131^I, thereby reducing the synthesis and secretion of thyroid hormones (8). Traditional dosing often involves a fixed range of 185–555 MBq (5–15 mCi), which overlooks individual patient differences, potentially leading to suboptimal outcomes or side effects (9). The dosing formula used is [Z × thyroid size (g) × 100]/24-hour iodine uptake rate (RAIU), where Z is the planned Bq or μCi per gram of thyroid tissue, ranging from 3.7 to 7.4 MBq (70-150 μCi) (10). However, these traditional methods have significant limitations. First, they highly depend on the accurate assessment of thyroid weight, while ultrasound measurement of thyroid volume has considerable inter-operator variability and measurement errors (11). Second, the existing formulas fail to adequately incorporate multiple clinical and pathophysiological factors affecting the biological effects of ^131^I, such as thyroid hormone levels, thyroid stimulating antibody (TSAb) titers, thyroid blood flow status, and individual metabolic differences (12). This leads to significant heterogeneity in patient outcomes after treatment, with some patients unable to achieve complete elimination (defined as the restoration of normal thyroid function), while others may develop permanent hypothyroidism, requiring lifelong thyroid hormone replacement therapy (13). Studies have shown that currently about 10%-30% of patients fail to achieve complete elimination after receiving ^131^I therapy, and the incidence of hypothyroidism can reach 20%-40% in the first year after treatment, increasing year by year over time (14, 15). This uncertainty in outcomes poses challenges for clinical decision-making, highlighting the urgent need for more precise dose prediction models to optimize treatment effects.

In recent years, with the rapid development of artificial intelligence (AI) technology in the medical field, machine learning (ML) algorithms have provided new solutions for complex medical prediction problems (16). Random Forest Regression (RFR), as an ensemble learning algorithm, effectively handles high-dimensional data, nonlinear relationships, and interactions between variables by constructing multiple decision trees and aggregating their prediction results, while also possessing strong anti-overfitting capabilities (17). In the medical field, RFR has been widely applied in various aspects such as disease diagnosis, prognosis prediction, and treatment response assessment (18). For example, in oncology, the RFR model successfully predicted the treatment response of Hepatocellular Carcinoma to transarterial radioembolization, achieving an accuracy rate of 79.6% (19); in the study of neurological diseases, ML models based on Random Forest (RF) showed good performance in predicting the prognosis of Leukemia, although its clinical translation still faces methodological challenges (20); in the cardiovascular field, RFR has been used to predict the risk of metabolic syndrome, with an area under the receiver operating characteristic curve (AUC) reaching 0.89 (21). These applications confirm the advantages of RFR in handling complex biomedical data.

The application of RFR in predicting the dosage of ^131^I treatment for GH has significant theoretical and practical implications. Firstly, RFR can integrate multidimensional predictive variables, including demographic characteristics (such as age and gender), clinical parameters (such as thyroid hormone levels, thyroid volume, and iodine uptake rate), immunological indicators (such as TSAb titer), and treatment-related factors (such as previous treatment history). By analyzing the complex relationships between these variables and treatment outcomes, the RFR model is expected to overcome the limitations of traditional formulas and achieve more personalized dosage calculations (17). Secondly, RFR has the capability to handle missing data and imbalanced datasets, which is particularly important for retrospective medical data modeling (22). In addition, the RF algorithm can assess variable importance, helping to identify key factors affecting the efficacy of ^131^I treatment.

It is worth noting that the development of prediction models based on RF must adhere to rigorous methodological standards, including appropriate sample size assurance, feature selection processes, model validation strategies, and performance evaluation metrics. In recent years, multiple studies have emphasized that ML models in clinical applications need to balance predictive accuracy and interpretability (23–25). For example, in the diagnostic models for acute myocardial infarction, combining interpretative AI techniques such as SHAP (SHapley Additive exPlanations) can enhance model transparency and promote clinical acceptance (26). Similarly, in predicting the dosage of ^131^I treatment for GH, the interpretability of the model helps clinicians understand the basis of predictions, thereby more effectively integrating model results into treatment decisions.

In summary, although ^131^I treatment for GH has good efficacy, the lack of precision in the dose calculation method leads to significant variability in treatment outcomes. The RFR model, as a powerful ML tool, can integrate multi-source heterogeneous data and capture the complex mapping relationship between variables and treatment response, promising more accurate individualized dose prediction. By constructing and validating a RF-based ^131^I treatment dose prediction model, it can not only improve the effectiveness and safety of GH treatment and reduce the occurrence of complications such as hypothyroidism, but also provide a new paradigm for precision medicine in autoimmune thyroid diseases.

## 2 Methods

### 2.1 Study design

A retrospective cohort study was conducted utilizing comprehensive clinical data from 975 adult patients diagnosed with GH who achieved remission following the administration of ^131^I therapy between January 25, 2015, and August 8, 2023. To ensure that the model accurately identifies features associated with successful treatment outcomes, the study cohort was exclusively composed of patients who achieved remission, defined as either euthyroidism or hypothyroidism, after a single dose of ^131^I. Consequently, the total iodine dose (TID) in this study is characterized as the “successful curative dose,” referring to the specific activity of ^131^I administered to a patient with a particular clinical profile that resulted in confirmed remission. Although this methodology is based on historical clinical decisions, the exclusion of treatment failures ensures that the model does not incorporate insufficient dosing strategies.

The inclusion criteria were as follows: a confirmed diagnosis of GH, validated through clinical symptoms, thyroid function tests, and autoantibody detection; and receipt of a single dose of ¹³¹I therapy resulting in either complete remission or the onset of hypothyroidism. The exclusion criteria included: (1) patients who remained hyperthyroid (treatment failure); (2) women who were pregnant or lactating; (3) individuals with a history of thyroid surgery; (4) patients unable to comply with regular follow-up schedules; (5) patients diagnosed with granulocyte deficiency and/or liver failure; and (6) individuals with a history of malignancies.

A retrospective cohort study design will be predominantly utilized to examine the predictive factors and TID for GH. This methodological approach facilitates the evaluation of outcomes in patients who have previously undergone treatment and possess documented follow-up data, thereby reducing selection bias by incorporating consecutive patient records when available. Baseline clinical, laboratory, and imaging data, documented prior to the initiation of ^131^I therapy, will be systematically extracted from medical records. Follow-up data regarding the recovery of thyroid function or the onset of hypothyroidism post-therapy will be meticulously recorded at predefined intervals. These intervals will generally extend over a period of at least 6 to 12 months, or potentially longer, to comprehensively evaluate both short-term efficacy and long-term outcomes, such as the onset of permanent hypothyroidism. The primary endpoints of interest will encompass the achievement of euthyroidism (normal thyroid function) or hypothyroidism, thereby classifying patients into “remission” (euthyroid or hypothyroid) and “non-remission” (persistent hyperthyroidism, partial remission, or no change) categories. The experimental procedure comprised four principal stages: data preparation, variable engineering, model training and prediction, and validation analysis. The model was developed to predict the TID by estimating the available clinical indicators. The predictive outcome of the applied models was defined as the TID required to achieve a remission status in patients with GH.

### 2.2 Patients’ preparation and I-131 therapy

The procedure and its associated precautions were comprehensively communicated to all patients, with particular emphasis placed on the necessity of adhering to a low-iodine diet and avoiding medications containing iodide for a duration of 7 to 14 days prior to treatment. Furthermore, ATD were required to be discontinued at least one week before the administration of ^131^I therapy. Laboratory evaluations included the measurement of thyroid-stimulating hormone (TSH), triiodothyronine (T3), thyroxine (T4), free triiodothyronine (FT3), free thyroxine (FT4), thyroglobulin antibody (TgAb), thyroid peroxidase antibody (TPOAb), and thyrotropin receptor antibody (TRAb) 1 to 2 days prior to the ^131^I therapy.

Our hospital employs a calculated dosage method to determine the I-131 treatment dose, administered using a fully automated ^131^I dispensing machine, based on the formula:


$$I-131\;treatment\;dose\;\left(\mu Ci\right)=\;\frac{Dose\;per\;gram\;of\;thyroid\;tissue\;\left(uCi\right)\times \;Thyroid\;weight\;\left(g\right)}{24h\;thyroid\;uptake\;rate\;of\;I-131\;\left(\%\right)}$$

According to their clinical condition, three expert nuclear medicine physicians prescribed the iodine dose per gram of thyroid tissue (IDPG) for each patient, generally between 70-120 μCi/g.

### 2.3 Assessment of therapeutic efficacy

Patients were monitored for a period ranging from six months to one year following the administration of ^131^I therapy. The therapeutic efficacy was evaluated using established criteria (27). Euthyroidism was characterized by the absence of clinical manifestations of hyperthyroidism and the presence of normal serum concentrations of FT3, FT4, and TSH. Hypothyroidism was diagnosed in patients presenting with clinical symptoms or signs of hypothyroidism, or in their absence, if serum FT3 and FT4 concentrations were below the normal range and TSH concentrations were elevated. Partial remission was identified by a reduction in hyperthyroidism symptoms, partial resolution of clinical signs, and a decrease in serum FT3 and FT4 concentrations, although these did not normalize. Ineffective responses was defined by either no significant improvement or a worsening of hyperthyroidism symptoms and signs, with no reduction in serum FT3 and FT4 concentrations. Outcomes of euthyroidism or hypothyroidism were classified as “remission,” whereas partial remission and ineffective responses were categorized as “non-remission.”

### 2.4 Candidate predictors

A comprehensive set of potential predictors, encompassing demographic, clinical, immunological, and treatment-related factors, will be systematically gathered to encapsulate the multifactorial nature of ^131^I therapy outcomes in GH. These variables will be standardized in terms of measurement methods, units, and timing of collection (primarily pre-treatment) to ensure consistency and comparability of data across the cohort.

#### 2.4.1 Demographic variables

##### 2.4.1.1 Age

Recorded in years at the time of initial ^131^I therapy.

##### 2.4.1.2 Gender

Documented as male or female, with a code of “1” representing male and a code of “2” denoting female.

#### 2.4.2 Clinical parameters

##### 2.4.2.1 Thyroid hormones and TPOAb

These were measured using the UniCel DxI 800 Access Immunoassay System with a chemiluminescence method: TSH: 0.56-5.91 μIU/mL; T3: 0.92-5.91 nmol/L; T4: 69.71-163.95 nmol/L; FT3: 3.53-7.37 pmol/L; FT4: 7.98-16.02 pmol/L; TPOAb:<9.0 IU/mL.

##### 2.4.2.2 TRAb

Measured using the UniCel DxI 800 Access Immunoassay System, with a reference range of 0-1.75 IU/L.

##### 2.4.2.3 Evaluation of radioactive iodine uptake

The study assessed thyroid iodine uptake rates utilizing I-131, supplied by Nanning Atomic High-throughput Isotope Co., Ltd. Prior to the evaluation, patients were instructed to abstain from iodine-containing foods and medications for a period of 2 to 4 weeks. On the day of assessment, patients ingested sodium iodide-131, with doses ranging from 2 to 10 μCi, in a fasting state in the morning. Following ingestion, patients remained fasting for an additional 2 hours. Radioactivity measurements of the thyroid region were subsequently conducted at 3 hours and 24 hours post-administration using the NM-6110 thyroid function measuring instrument. The effective half-life (Teff) was determined from the sequential I-131 uptake measurements. Teff is defined as the duration required for the activity within the thyroid gland to decrease to 50% of its initial value, considering the combined effects of physical decay and biological clearance.

##### 2.4.2.4 Thyroid weight

After intravenous injection of ^99m^TcO4^-^ (2-5mCi), thyroid imaging was performed 15–20 minutes later. The patient was positioned supine with a pillow under the shoulder and neck to hyperextend the neck and fully expose the thyroid. Images were collected using the Discovery NM/CT 670, equipped with a low-energy general collimator, a matrix size of 256×256, an energy peak of 140keV, a window width of ±10%, and a collection count of 300k. The region of interest (ROI) was delineated in the blue-purple interface of the thyroid color image using Xeleris post-processing software to obtain the thyroid area, height, and weight.

#### 2.4.3 Treatment-related factors

##### 2.4.3.1 History of ATD therapy

The variable “ATD” represents the history of ATD usage, where a code of “0” signifies no prior use and a code of “1” indicates a positive history of use.

##### 2.4.3.2 Administered ^131^I dosage

This refers to the prescribed dose of ^131^I in millicuries (mCi), as well as the IDPG measured in megabecquerels per gram (MBq/g). The variable “IDPG” categorizes the iodine dose per gram of thyroid tissue, with a code of “1” denoting small doses (70-90 μCi/g) and a code of “2” indicating large doses (91-120 μCi/g).

##### 2.4.3.3 Course of disease

This refers to the duration of GH prior to ^131^I therapy. The variable “Disease_course” is defined by the length of the illness, with a code of “0” indicating a duration of two years or less, and a code of “1” denoting a duration exceeding two years.

### 2.5 Missing data handling

Prior to model development, we conducted an assessment of the dataset’s completeness across all collected variables. Notably, missing values were observed in the TPOAB (6.9%) and TRAB (15.0%) variables. Patients with entirely missing outcome data (TID) were excluded from the analysis. To address missing covariates without reducing the sample size, we employed the MissForest algorithm, a non-parametric imputation method based on RF, which enables the estimation of missing values in mixed-type data (22).

To avoid data leakage, data splitting was executed before imputation. The imputation model was trained solely on the development set (n = 633, spanning from 25 January 2015 to 25 January 2021) and subsequently applied to impute missing values in the temporal validation set (n = 342, covering the period from 26 January 2021 to 8 August 2023).

### 2.6 Prediction model

A RFR model is proposed for development to predict the effective TID for individual patients with GH. This ensemble ML algorithm has been selected due to its robustness in managing high-dimensional datasets, its capability to capture intricate nonlinear relationships, and its intrinsic resistance to overfitting, rendering it particularly suitable for medical prediction tasks (28, 29).

The RFR model functions by generating numerous decision trees during the training phase, with each tree constructed from a bootstrap sample of the training dataset. In the context of regression tasks, the ultimate prediction is derived from aggregating the predictions of all individual trees, typically through averaging, which serves to reduce the variance associated with individual decision trees. This approach enables the model to effectively incorporate diverse data types, encompassing both continuous and categorical variables, without necessitating extensive preliminary assumptions regarding their distributions.

#### 2.6.1 Key technical details of the RFR model include

##### 2.6.1.1 Model inputs

The model is designed to incorporate a comprehensive set of patient-specific variables, which include demographic information (such as age and sex), clinical parameters (including FT3, FT4, TSH, thyroid weight, and RAIU), immunological markers (such as TRAb, and TPOAB), and treatment-related factors (such as prior ATD use and IDPG).

##### 2.6.1.2 Model output

The principal output of the model is a continuous variable that estimates the TID required for an individual patient, with the aim of achieving either euthyroidism or controlled hypothyroidism.

##### 2.6.1.3 Hyperparameters

Hyperparameter tuning was rigorously performed on the training set using RandomizedSearchCV with 5-fold cross-validation. The optimization process targeted the following parameters:

n_estimators: range [100, 200, 300, 500, 1000]
max_depth: range [10, 20, 30, None]
min_samples_split: range [2, 5, 10]
min_samples_leaf: range [1, 2, 4]

The parameters for the model were set as follows: the number of trees (n_estimators) was 100; the minimum number of samples required to split an internal node (min_samples_split) was 2; the minimum number of samples required at a leaf node (min_samples_leaf) was 1; the minimum impurity decrease required for a split (min_impurity_decrease) was 0.0; the maximum number of features considered for splitting a node (max_features) was set to the square root of the total number of features; the maximum depth of the tree (max_depth) was unrestricted; and the criterion used for measuring the quality of a split was the Friedman mean squared error (friedman_mse).

##### 2.6.1.4 Ensemble learning

The ensemble characteristic of RF enables it to mitigate the biases of individual trees through averaging, resulting in a more robust and precise overall prediction. Furthermore, it has the inherent capability to identify feature interactions, which is advantageous when dealing with complex medical datasets.

The RFR model’s ability to handle complex nonlinear relationships among predictors and its robustness against overfitting are particularly valuable in the medical domain, where patient responses to therapy are often multifactorial and intricate.

### 2.7 Statistical methods

**Figure 1:**
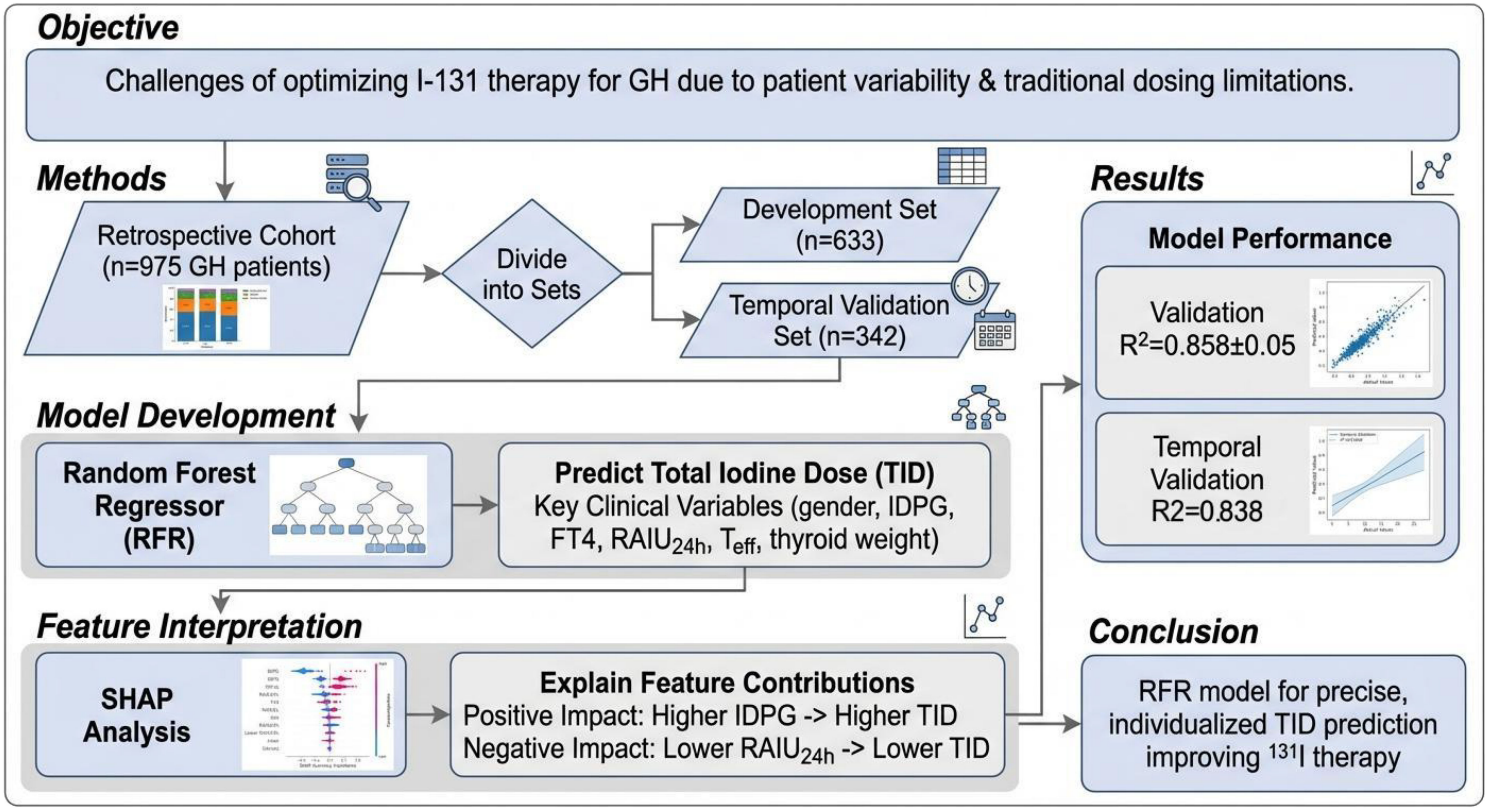
Research flowchart.

All statistical analyses were conducted using R version 4.2.3 and Python version 3.11.4 on the collected dataset. The Shapiro-Wilk test was employed to assess the normality of continuous variables. For variables exhibiting a normal distribution, either analysis of variance (ANOVA) was applied for comparisons involving more than two groups, or the t-test was used to evaluate statistical significance. In instances where the data did not adhere to a normal distribution, the Kruskal-Wallis test was applied for significance testing. For categorical variables, statistical significance was determined using either the chi-square test or Fisher’s exact test. The cohort, consisting of 975 patients, was divided into a development set (n = 633, spanning from 25 January 2015 to 25 January 2021) and a temporal validation set (n = 342, covering the period from 26 January 2021 to 8 August 2023). The TID was designated as the outcome variable. Independent risk factors were identified using the least absolute shrinkage and selection operator (LASSO) Gaussian regression analysis, and a machine learning prediction model was developed using RFR. LASSO Gaussian regression analysis is a linear regression technique that employs L1 regularization, making it particularly suitable for modeling continuous outcome variables that adhere to a Gaussian distribution. The model’s discriminative capacity was assessed by R-squared, mean squared error, and explained variance score. A P value below 0.05 was deemed statistically significant (**Figure 1**).

## 3 Results

### 3.1 General information

The baseline characteristics of the study cohort, stratified by the development and temporal validation samples, are presented in **Table 1**. The median age was 36 (Q1–Q3: 29–43) years in the overall population, with comparable distributions between the development (36 [28–43]) and validation (37 [29–43]) groups (p = 0.599). Gender distribution was balanced, with 55.3% females overall and no significant difference between samples (p = 0.794). Among clinical and biochemical variables, all continuous measures—including TID (4.95 [4.30–5.72]), FT3 (23 [16–31]), FT4 (54 [43–64]), FT3/FT4 ratio (0.44 [0.37–0.50]), RAIU at 3h (66 [53–78]) and 24h (84 [76–91]), Teff (5.64 [5.14–6.10]), thyroid weight (46 [38–56]), urine iodine (63 [59–69]), TPOAb (349 [72–690]), and TRAb (16 [11–22])—showed no statistically significant differences between the development and validation samples (all p > 0.05). Similarly, categorical variables such as history of ATD usage (55.2% yes), disease course over 2 years (6.8% yes), and IDPG dosage groups (69.1% in 70–90 μCi/g) were well balanced across both samples (all p > 0.20), indicating that the two groups were comparable at baseline.

**Table 1:** Comparison of general data between development set and temporal validation set patients.

Characteristic	Group			p-value
	Overall N = 975	Development set N = 633	Temporal validation set N = 342	
Study setting				
Data collection period		25 January 2015 to 25 January 2021	26 January 2021 to 8 August 2023	
Study design		Retrospective		
Age, Median (Q1, Q3)	36 (29, 43)	36 (28, 43)	37 (29, 43)	0.599^1^
Gender, n (%)				0.794^2^
female	539 (55.3%)	348 (55.0%)	191 (55.8%)	
male	436 (44.7%)	285 (45.0%)	151 (44.2%)	
TID, Median (Q1, Q3)	4.95 (4.30, 5.72)	4.93 (4.29, 5.70)	5.00 (4.30, 5.80)	0.567^1^
FT3, Median (Q1, Q3)	23 (16, 31)	22 (16, 31)	24 (16, 31)	0.797^1^
FT4, Median (Q1, Q3)	54 (43, 64)	54 (43, 63)	55 (44, 64)	0.877^1^
FT3/FT4, Median (Q1, Q3)	0.44 (0.37, 0.50)	0.44 (0.37, 0.50)	0.44 (0.37, 0.50)	0.492^1^
RAIU3h, Median (Q1, Q3)	66 (53, 78)	65 (52, 79)	67 (54, 77)	0.553^1^
RAIU24h, Median (Q1, Q3)	84 (76, 91)	84 (75, 91)	85 (76, 91)	0.291^1^
Teff, Median (Q1, Q3)	5.64 (5.14, 6.10)	5.64 (5.13, 6.10)	5.64 (5.19, 6.08)	0.921^1^
Thyroid weight, Median (Q1, Q3)	46 (38, 56)	46 (38, 55)	46 (39, 57)	0.318^1^
Urine iodine, Median (Q1, Q3)	63 (59, 69)	63 (59, 70)	62 (59, 68)	0.163^1^
TPOAB, Median (Q1, Q3)	349 (72, 690)	345 (68, 690)	353 (74, 680)	0.530^1^
TRAB, Median (Q1, Q3)	16 (11, 22)	16 (11, 22)	17 (12, 23)	0.263^1^
The history of ATD usage, n (%)				0.210^2^
No	437 (44.8%)	293 (46.3%)	144 (42.1%)	
Yes	538 (55.2%)	340 (53.7%)	198 (57.9%)	
Disease course over 2years, n (%)				0.968^2^
No	909 (93.2%)	590 (93.2%)	319 (93.3%)	
Yes	66 (6.8%)	43 (6.8%)	23 (6.7%)	
IDPG, n (%)				0.603^2^
70–90 μCi/g	674 (69.1%)	434 (68.6%)	240 (70.2%)	
91–120 μCi/g	301 (30.9%)	199 (31.4%)	102 (29.8%)	

^1^Wilcoxon rank sum test.

^2^Pearson’s Chi-squared test.

### 3.2 Variable selection

**Figure 2:**
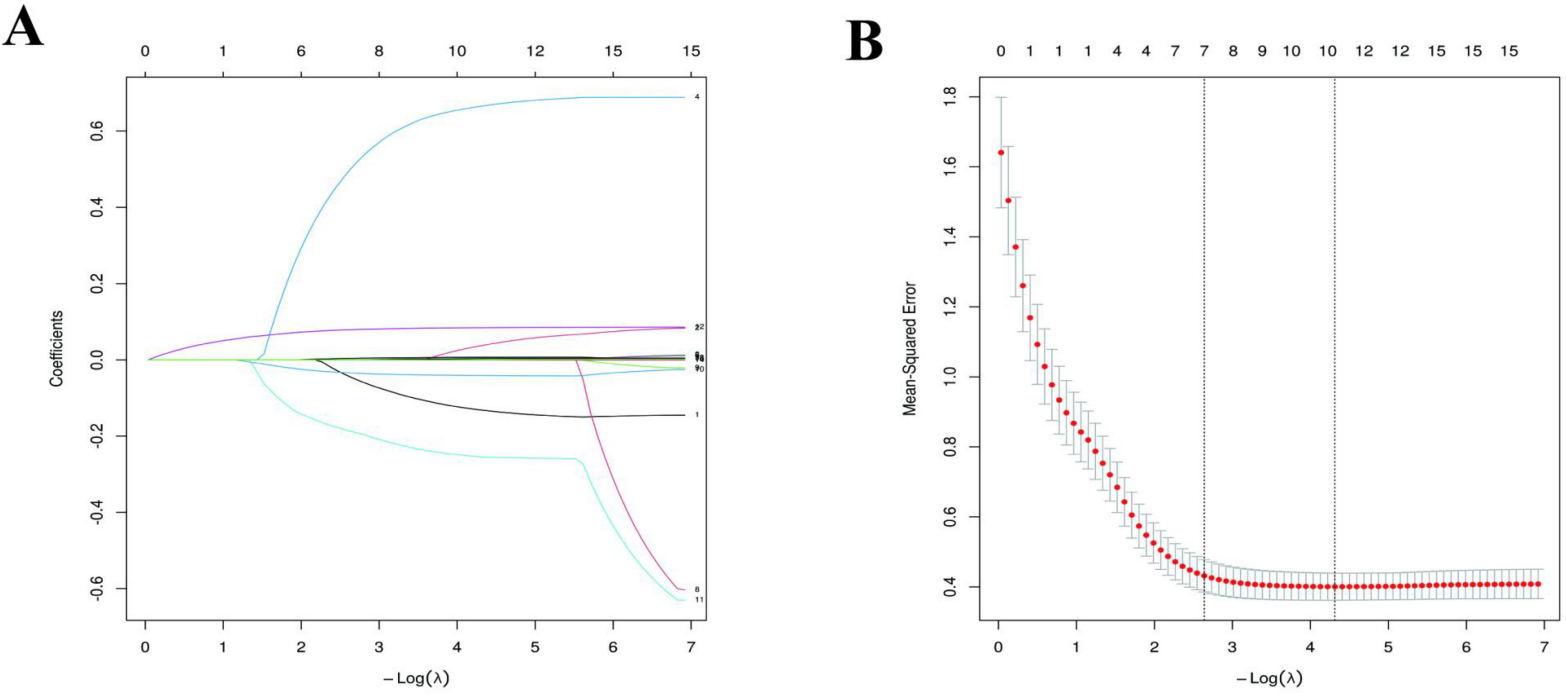
LASSO-Gaussian regression analysis for selecting predictive variables in development set; **(A)** illustrates optimal variable selection via 10-fold cross-validation. **(B)** displays the coefficient curve of 15 clinical features.

Utilizing LASSO-Gaussian regression analysis, we optimized and selected a total of 15 variables. The optimal value was determined by the minimum 10-fold cross-validation error within one standard error (1SE). The λ value corresponding to the minimum standard error of distance was identified as 0.067, resulting in the selection of seven predictive variables with non-zero coefficients. These variables are Gender, IDPG, FT4, RAIU24h, Teff, and Thyroid weight, as detailed in **Table 2** and illustrated in **Figures 2A, B**.

**Table 2:** Variable selection results using LASSO-Gaussian regression analysis coefficients in development set.

Name	MSE	1SE
(Intercept)	4.645	4.54
Gender	-0.132	-0.046
The history of ATD usage	0.037	0.0
Disease course over 2 years	0.0	0.0
IDPG	0.666	0.502
Age	0.003	0.0
FT3	0.0	0.0
FT4	0.007	0.004
FT3/FT4	0.0	0.0
RAIU3h	0.0	0.0
RAIU24h	-0.041	-0.034
Teff	-0.255	-0.188
Thyroid weight	0.084	0.079
Urine iodine	0.003	0.0
TPOAB	0.0	0.0
TRAB	0.0	0.0

### 3.3 Prediction model construction and evaluation

**Figure 3:**
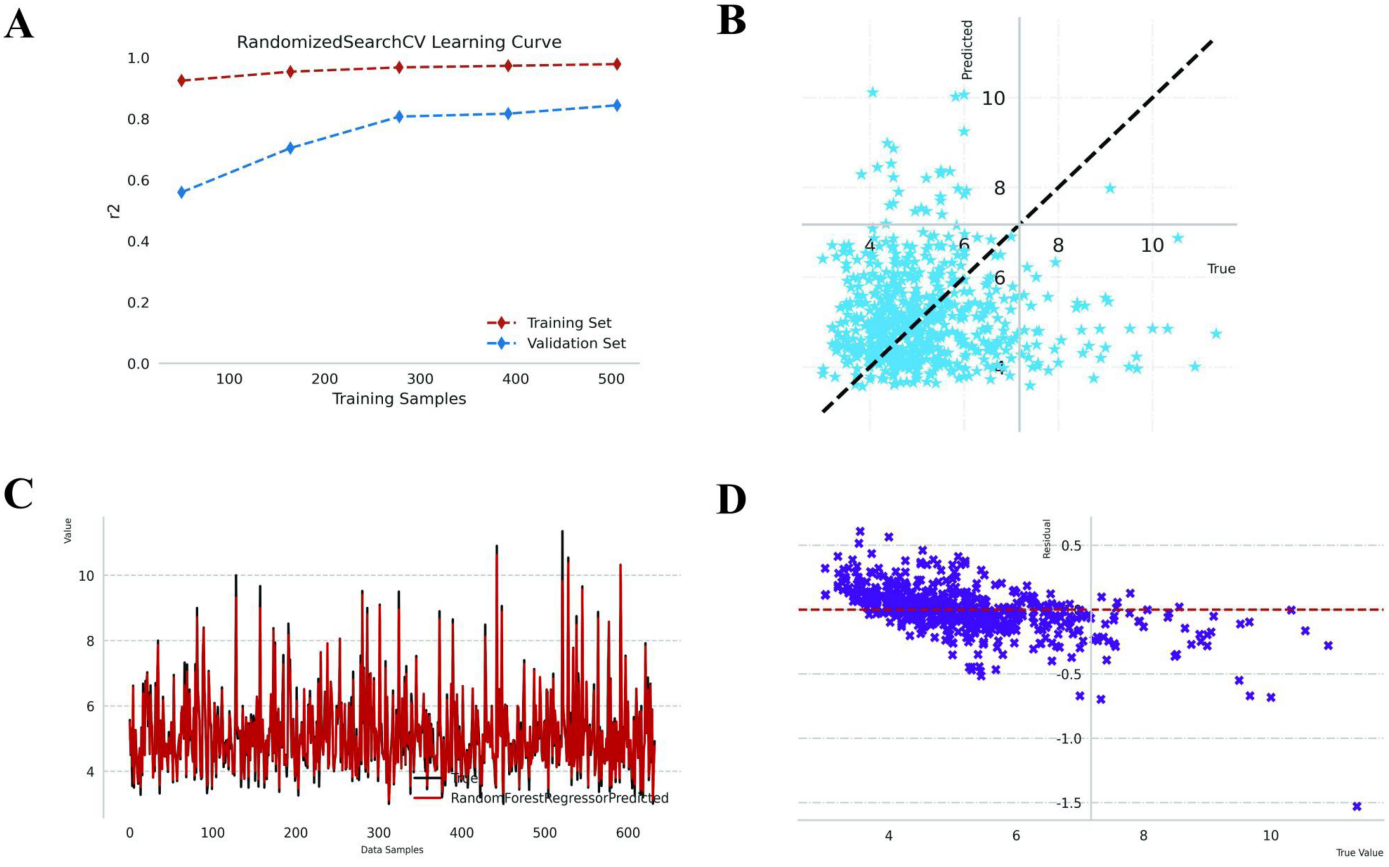
Performance metrics of the RFR. **(A)** Learning curves displaying R² scores for training (brown) and validation (blue) sets. **(B)** Scatter plot comparing Predicted vs. Actual TID (mCi) (N = 342); blue stars align with the diagonal, indicating high accuracy. **(C)** Line plot contrasting True (black) and Predicted (red) TID values (mCi) across test samples. **(D)** Residual plot showing the difference between predicted and actual values (mCi); residuals (purple crosses) are centered around zero (red dashed line), indicating minimal bias. Abbreviation: TID, Total Iodine Dose.

In this study, the RFR algorithm was employed for regression analysis, with TID as the outcome variable. The model incorporated the following variables: Gender, IDPG, FT4, RAIU24h, Teff, and Thyroid weight. The cohort, consisting of 975 patients, was divided into a development set (n = 633, spanning from 25 January 2015 to 25 January 2021) and a temporal validation set (n = 342, covering the period from 26 January 2021 to 8 August 2023). The remaining samples were utilized as the training set for 5-fold cross-validation. The final model achieved an R-squared value of 0.858 ± 0.05 on the validation set and 0.838 on the temporal validation set (**Table 3**). **Figure 3A** illustrates the learning curve for RandomizedSearchCV. The R² value for the development set, represented by the red dashed line, is consistently high and stable as the number of training samples increases, indicating robust model performance on the training data. Conversely, the R² value for the validation set, depicted by the blue dashed line, shows an upward trend with an increasing number of training samples. **Figure 3B** presents a scatter plot of predicted values versus actual values, with the dashed line representing the ideal scenario where predictions perfectly align with actual values. The concentration of blue star points near this line suggests that the model exhibits predictive capability. Nonetheless, the presence of points that significantly deviate from the dashed line indicates the occurrence of substantial errors in certain predictions. **Figure 3C** illustrates the variation in the model’s predicted values (depicted by the red line) and the actual values (represented by the black line) as the data samples change. The figure demonstrates that the predicted and actual values generally exhibit a similar trend, suggesting that the model effectively captures the overall pattern of the data. However, there are discernible discrepancies at certain peaks, indicating that the model may struggle with accurately predicting extreme values. **Figure 3D** presents a residual plot, which displays the relationship between the model’s residuals (calculated as the difference between predicted and actual values) and the actual values. Ideally, the residuals should be randomly distributed around the zero line (indicated by the red dashed line in the figure) without any discernible pattern. In this plot, the residuals (marked by purple crosses) are predominantly clustered near the zero line, although some larger outliers are present. Additionally, the dispersion of residuals appears to increase with higher actual values, suggesting a potential decline in the model’s predictive accuracy for larger actual values.

**Table 3:** Summary of the development set and temporal validation set results.

Cohort	R-squared	Mean Squared Error (MSE)	Explained Variance (EV)
Development set	0.858 ± 0.05	0.235 ± 0.095	0.859 ± 0.05
Temporal validation set	0.838	0.304	0.84

### 3.4 Interpretability analysis

**Figure 4:**
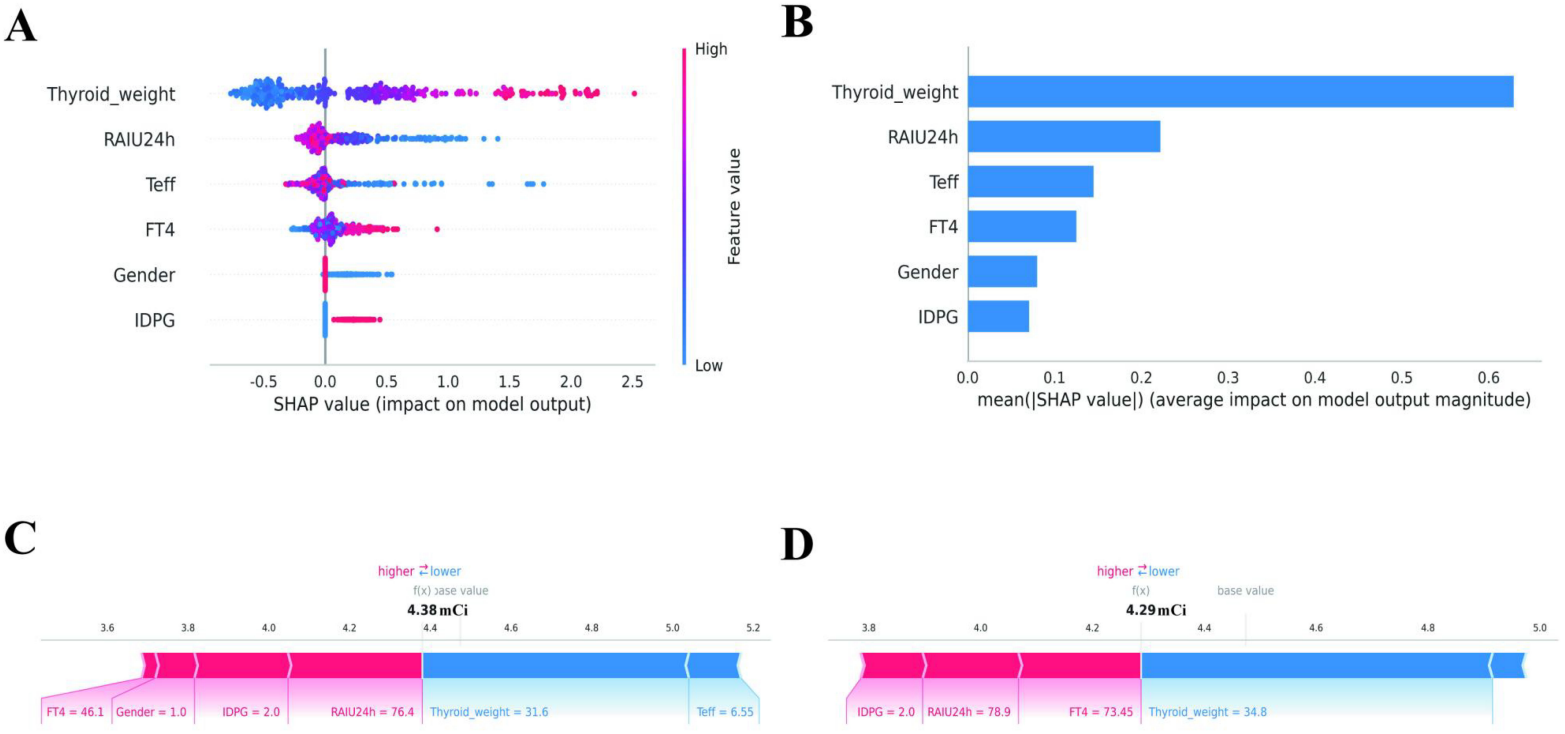
Feature importance and contribution analysis. **(A)** SHAP summary plot illustrating global feature importance. Each dot represents a patient sample. Color indicates the feature value: Red = High value, Blue = Low value. For example, higher Thyroid Weight (red dots) is associated with positive SHAP values (increased dose prediction). Features are ranked by the mean absolute SHAP value. **(B)** Bar chart showing the mean absolute SHAP values, quantifying the average impact of each feature on the model output (units correspond to the feature’s scale). **(C, D)** Local SHAP force plots for two individual predictions. Red bars indicate features that push the prediction higher (increase dose), while blue bars push it lower (decrease dose). The length of the bar represents the magnitude of the contribution.

The SHAP summary plot elucidates the aggregate contribution of features to the predictive model developed for thyroid-related outcomes. This model was constructed utilizing clinical data, including variables such as thyroid weight (threshold of 45.58g), RAIU24h (threshold of 83.74%), gender (threshold for female), IDPG (threshold of 70-90 μCi/g), FT4 (threshold of 54.35 pmol/L), and Teff (threshold of 5.64 d). Prior to the modeling process, feature values were standardized. SHAP values were computed to assess the incremental contribution of each feature to individual predictions. In the plot, each point represents an individual sample, with colors ranging from blue (indicating low feature values) to red (indicating high feature values). Features are organized in descending order based on their mean absolute SHAP values to illustrate their relative importance. A statistical summary of feature importance is presented as the mean ± standard deviation of the absolute SHAP values of the samples, as depicted in **Figures 4A, B**. Thyroid weight: The distribution of data points is broad, with a predominance of red points on the positive side, suggesting that larger thyroid weights significantly enhance model predictions, indicating a positive contribution. Conversely, the presence of blue points on the negative side implies that lower thyroid weights tend to decrease predictions. Overall, this feature exerts the most substantial impact on the model. RAIU24h: A greater number of red points are observed on the negative side, whereas blue points are more frequently located on the positive side. This pattern indicates that high RAIU24h values generally lead to a reduction in predictions, signifying a negative contribution, while low values tend to increase predictions, indicating a positive contribution. Teff, FT4: The distribution of points is of moderate width, with some correlation between color and position. This suggests that these features exert a moderate influence on the model and may exhibit partial nonlinearity. Gender, IDPG: The majority of points are situated close to zero, with no significant bias towards either side, indicating that these features have a relatively minor or unstable impact on model predictions.

To elucidate the model’s decision-making process at the individual level, a SHAP force plot was generated (**Figure 4C**). Using a representative sample with a predicted value of 4.38 mCi as an example, the visualization indicates that RAIU24h = 76.4% and IDPG (91-120 μCi/g) were the primary positive drivers elevating the prediction. In contrast, Thyroid_weight (31.6 g) and Teff = 6.55d served as negative contributors, reducing the model output. This illustrates the model’s capability to balance conflicting clinical factors for precise prediction. **Figure 4D** illustrates a representative case with a final predicted value of 4.29 mCi. The SHAP force plot reveals the interaction between conflicting features: on one hand, indicators of severe thyrotoxicosis, specifically markedly elevated FT4 (73.45 pmol/L) and RAIU24h (78.9%), acted as positive drivers, increasing the prediction. However, this effect was significantly mitigated by the Thyroid_weight (34.8 g), which the visualization identifies as the dominant negative contributor in this instance, effectively diminishing the impact of high hormone levels and resulting in a lower predicted outcome. This underscores the model’s ability to incorporate complex, multidimensional clinical variables that extend beyond basic linear correlations. In Case A (4.38 mCi), the data illustrate how RAIU increases the dosage. Conversely, Case B (4.29 mCi) exemplifies that despite a high RAIU, a particular thyroid weight can lead to a reduction in the predicted dosage.

## 4 Discussion

This study successfully developed and validated a RFR model designed to predict the TID for patients diagnosed with GH who have achieved remission. The model demonstrated robust predictive performance, achieving an R-squared value of approximately 0.858 ± 0.05 on the validation set and 0.838 on the temporal validation set, indicating its strong capability to explain the variance in ^131^I dosage. Key predictive variables identified through this model included patient Gender, IDPG, FT4 levels, RAIU24h, Teff, and Thyroid weight. Furthermore, the application of SHAP values provided crucial interpretability, offering insights into how each feature contributes to the model’s predictions, thereby enhancing clinical understanding and potential for personalized treatment strategies. This interpretability is vital for gaining clinical acceptance and fostering trust in AI-driven medical decision support tools.

The findings of this study significantly advance the landscape of ^131^I dose prediction for GH, building upon and distinguishing itself from prior research. Traditionally, ^131^I dosing methods have relied on empirical formulas that primarily consider thyroid gland size and RAIU (30). While these traditional approaches offer a practical foundation, they often exhibit limitations, including variability due to operator measurement errors and an incomplete incorporation of a wide array of biological and clinical factors (31, 32). Studies utilizing such formulas often yield moderate success rates and demonstrate variability in treatment outcomes (31). For instance, a study involving 970 GH patients indicated that thyroid mass, ^131^I dosage, thyroid hormone levels, and the presence of thyroid murmurs independently influenced therapy efficacy, highlighting the need for personalized parameters (32). However, these methods struggle with the complex, non-linear relationships inherent in biological systems and typically cannot evaluate a large number of predictors simultaneously (33).

In contrast, the current RFR model represents a substantial methodological leap by integrating a comprehensive set of patient-specific clinical, biochemical, and immunological variables, thereby capturing the multifactorial nature of treatment response more effectively. ML algorithms, such as neural networks and support vector machines, have demonstrated their ability to process multidimensional data and model complex, non-linear relationships to improve dose prediction and outcome classification (34, 35). The superior performance of the RFR model, evidenced by its high R-squared values, aligns with recent literature advocating for the use of ensemble learning methods, particularly RF, to integrate multifaceted clinical and biochemical parameters for enhanced therapeutic dose prediction accuracy (33, 36). RF models are known for their robustness against overfitting and their capacity to handle large, complex, and heterogeneous datasets without extensive preprocessing, which is a significant advantage in medical research. Studies have shown that RF models can achieve high classification accuracy, often exceeding 90% in similar prediction tasks within hyperthyroidism patient cohorts (34). This study not only confirms the superiority of ML approaches but also provides an interpretable model, which is crucial for clinical adoption and understanding the underlying drivers of dose efficacy. The present findings strongly support and advance the viewpoint that ML, especially ensemble methods like RF, significantly enhances individualized dosimetry beyond the capabilities of classical empirical formulas, paving the way for more precise and personalized ^131^I therapy in GH.

Thyroid weight has long been recognized as a fundamental predictor in determining the success and dosing of ^131^I therapy for GH. The consistency of this factor across numerous studies underscores its central role in predicting outcomes, particularly regarding cure rates and the incidence of hypothyroidism (37–39). Recent literature, including a study on 325 GD patients, found that a smaller thyroid weight was significantly associated with successful radioiodine therapy (37). Similarly, a study involving 724 patients noted that successfully treated individuals had a smaller thyroid weight at presentation (38). Another more recent analysis confirmed thyroid volume as a significant independent predictor of radioactive iodine therapy efficacy, with smaller volumes correlating with better outcomes (40). A meta-analysis in 2022 further solidified the importance of patient characteristics, including thyroid size, in predicting ^131^I therapy failure in GD (41). This study reaffirms the established importance of thyroid weight as a critical determinant in ^131^I dosing and efficacy prediction.

RAIU24h is essential for assessing thyroid function and guiding ^131^I therapy, predicting treatment success and hypothyroidism risk. Lower RAIU24h levels increase early hypothyroidism risk and are the sole risk factor one year post-therapy (40, 42, 43). This study enhances its predictive role by using a RF model, capturing complex interactions with other clinical features that traditional models miss. The integration allows for a comprehensive analysis of RAIU24h’s interplay with clinical, biochemical, and immunological factors, as shown by SHAP analysis, which highlights its influence on the predicted ^131^I dose alongside factors like FT4 and thyroid weight.

FT4 levels are key indicators of hyperthyroidism severity and predictors of ^131^I therapy response. Studies show that lower FT4 levels correlate with successful radioiodine treatment and high remission rates, while higher levels suggest more severe disease and potential treatment challenges (37, 38, 44). The current study uses FT4 in a RF model to explore its complex interactions with other predictive factors, aligning with clinical insights. Unlike simpler models that view FT4 in isolation, the RFR model considers its interaction with factors like thyroid weight, RAIU, and immunological markers. In this model, FT4 influences predictions based on its numerical value: elevated FT4 levels lead to significantly higher predicted values, indicating that high FT4 levels elevate the prediction outcomes; conversely, lower FT4 levels result in decreased predicted values, suggesting that low FT4 levels adversely affect predictions. Overall, FT4 emerges as a pivotal feature within the model, exerting a significant bidirectional impact on predictions and demonstrating a positive correlation with the predicted outcomes. This integrative approach facilitates a more precise and individualized dose prediction by incorporating these elements.

Traditionally, gender has been viewed as a demographic factor rather than a predictor for ^131^I dosing in GH, despite its higher prevalence in females. Recent research, however, is examining sex-based differences in disease traits and treatment responses (45). While past studies often mentioned gender without recognizing it as a strong predictor, newer analyses suggest gender may influence prognosis, with factors like TPOAb affecting males differently (39). The present study distinctively integrates gender as a crucial predictive factor within its RFR model, thereby enhancing the personalization of ^131^I therapy. This advancement recognizes and quantifies the influence of sex-based differences on the optimal dosage of ^131^I, an aspect frequently neglected by previous models. The RFR model effectively captures the intricate gender-related influences on treatment by incorporating a range of clinical and immunological factors, thus facilitating more accurate dose predictions. Nevertheless, analysis of the SHAP plot reveals that the data points corresponding to gender are predominantly clustered around a SHAP value near zero, with minimal horizontal dispersion. This indicates that the average contribution of gender to the model’s output is relatively small, and its influence on predictions for individual samples is limited, both positively and negatively.

Incorporating the IDPG into advanced ML models for ^131^I therapy marks a significant innovation in dosimetry. Traditional dosing methods, which use fixed activity ranges or formulas based on thyroid weight and uptake, often overlook various clinical factors, leading to inconsistent outcomes. While some studies have considered IDPG in analyzing hypothyroidism risk, its use as a key predictor in ML models for determining effective curative doses is rare (37). A study highlighted ^131^I activity per gram of thyroid tissue” as a crucial independent predictor of ^131^I efficacy (40). This study’s RFR model innovatively incorporates IDPG as an input variable, categorizing it into “small” (70-90 μCi/g) and “large” (91-120 μCi/g) doses to enhance the prediction of optimal therapeutic doses based on patient-specific factors. This methodology improves the model’s capacity to discern subtle dose-response relationships, thereby enabling precise and personalized therapeutic recommendations that extend beyond conventional guidelines. By incorporating IDPG as a predictive feature, the model provides a refined, data-driven dosing strategy. It is noted that variables like IDPG and Thyroid Weight are integral to traditional dosing formulas. Therefore, the high predictive accuracy partially reflects the model’s ability to replicate the clinical decision-making process. However, the RFR model adds value by capturing non-linear interactions between these factors that linear formulas overlook.

The Teff is vital for understanding the absorbed radiation dose, combining both the radioisotope’s decay and its biological elimination. Historically difficult to measure, Teff has been underused in ^131^I dosing models for GH (46). Although research has aimed to optimize Teff estimation, its direct use in prediction models is rare. A study acknowledged Teff’s importance (45), and another study attempted to predict it using RAIU measurements (46). The current study innovatively includes Teff as a key feature in its RFR model, enhancing dose prediction by considering individual patient variability in radioiodine kinetics. This inclusion fills a gap in past research by integrating Teff into the main dose prediction algorithm, rather than calculating it separately. By incorporating Teff, the RFR model accounts for individual variations in iodine retention and elimination, crucial for determining the absorbed dose and therapeutic effectiveness. SHAP analysis showed Teff as a key negative factor in predicting the ^131^I dose, implying that a longer Teff may require a lower administered dose due to extended exposure. This precise integration of a dynamic pharmacokinetic parameter enhances the model’s ability to personalize ^131^I dosing, improving treatment outcomes.

Despite the significant advancements introduced by this RFR model, several limitations must be acknowledged that affect its generalizability and potential for broader clinical application. Firstly, the study predominantly utilized a retrospective cohort design. Although this approach is advantageous for assembling a substantial sample size and accessing documented follow-up data, it inherently presents risks of selection bias and incomplete data capture from existing medical records. Retrospective data may not adequately control for confounding variables or biases introduced during the data collection process over time. Furthermore, since the model was trained on patients who achieved remission, it predicts the sufficient dose for cure rather than the minimum effective dose, potentially reflecting only the historical dosing practices of the participating center. Secondly, the data sources, while from designated medical centers, may represent single or limited centers, which can restrict the model’s generalizability across diverse patient populations and varied healthcare settings. This geographical and demographic homogeneity could limit the model’s applicability to other regions or ethnic groups with different clinical practices or genetic predispositions. Thirdly, although the sample size of 975 patients is substantial, it might still be insufficient to capture the full spectrum of variability, especially for rare patient subgroups or less common long-term outcomes of ^131^I therapy. Furthermore, while rigorous techniques such as LASSO regression were used for variable selection, there remains a possibility that certain latent factors or unmeasured biomarkers influencing therapy outcomes were omitted from the model. Measurement errors, particularly in highly subjective assessments like thyroid volume estimation via ultrasound, can introduce inaccuracies into the model’s inputs, thereby affecting its overall predictive precision. Lastly, despite RF models generally being robust against overfitting, the risk persists, particularly when trained exclusively on retrospective clinical data that may contain specific patterns not representative of broader clinical realities. The lack of external validation across independent datasets is also a notable limitation, affecting the confidence in the model’s performance when applied to new, unseen patient cohorts.

## 5 Conclusion

This study successfully developed and validated a RFR model for predicting the TID in patients with GH. The model demonstrated robust predictive performance with an R-squared value of 0.838 on the temporal validation set. By identifying Gender, IDPG, FT4, RAIU24h, Teff, and Thyroid weight as key predictive variables, our RFR model offers a sophisticated approach to dose personalization. The innovative integration of these multi-dimensional features, coupled with the interpretability provided by SHAP values, allows for a more nuanced understanding of their complex interactions and individual contributions to treatment outcomes. The findings pave the way for more precise and individualized ^131^I therapy, potentially replicate and refine the successful dosing patterns.

## Data Availability

The original contributions presented in the study are included in the article/supplementary material. Further inquiries can be directed to the corresponding authors.
